# Hepatic transcriptomic signatures of statin treatment are associated with impaired glucose homeostasis in severely obese patients

**DOI:** 10.1186/s12920-019-0536-1

**Published:** 2019-06-03

**Authors:** Daniel Margerie, Philippe Lefebvre, Violeta Raverdy, Uwe Schwahn, Hartmut Ruetten, Philip Larsen, Alain Duhamel, Julien Labreuche, Dorothée Thuillier, Bruno Derudas, Céline Gheeraert, Hélène Dehondt, Quentin Dhalluin, Jérémy Alexandre, Robert Caiazzo, Pamela Nesslany, Helene Verkindt, François Pattou, Bart Staels

**Affiliations:** 1 Research & Development, Sanofi Aventis Deutschland GmbH, D-65926 Frankfurt, Germany; 2 Univ. Lille, INSERM, CHU Lille, Institut Pasteur de Lille, U1011 – EGID, F-59000 Lille, France; 3 Univ. Lille, Inserm, CHU Lille, Institut Pasteur de Lille, U1190 – EGID, F-59000 Lille, France; 4 Department of Biostatistics, CHU Lille, F-59000 Lille, France; 5 Department of General and Endocrine Surgery, CHU Lille, F-59000 Lille, France

**Keywords:** Statin, Human, Liver, Iatrogenic diabetes, Gene expression, Gene networks

## Abstract

**Background:**

Clinical data identified an association between the use of HMG-CoA reductase inhibitors (statins) and incident diabetes in patients with underlying diabetes risk factors such as obesity, hypertension and dyslipidemia. The molecular mechanisms however are unknown.

**Methods:**

An observational cross-sectional study included 910 severely obese patients, mean (SD) body mass index (BMI) 46.7 (8.7), treated with or without statins (ABOS cohort: a biological atlas of severe obesity). Data and sample collection took place in France between 2006 and 2016. Transcriptomic signatures of statin treatment in human liver obtained from genome-wide transcriptomic profiling of five different statin drugs using microarrays were correlated to clinico-biological phenotypes and also assigned to biological pathways and mechanisms. Patients from the non-statin-users group were matched to patients in the statin users group by propensity score analysis to minimize confounding effects from age, gender, parental familial history of diabetes, BMI, waist circumference, systolic and diastolic blood pressure and use of anti-hypertensive drugs as pre-specified covariates.

**Results:**

We determined the hepatic, statin-related gene signature from genome-wide transcriptomic profiling in severely obese patients with varying degrees of glucose tolerance and cardio-metabolic comorbidities. One hundred and fifty seven patients on statin treatment in the matched cohort showed higher diabetes prevalence (OR = 2.67; 95%CI, 1.60–4.45; *P* = 0.0002) and impairment of glucose homeostasis. This phenotype was associated with molecular signatures of increased hepatic de novo lipogenesis (DNL) via activation of sterol regulatory element-binding protein 1 (SREBP1) and concomitant upregulation of the expression of key genes in both fatty acid and triglyceride metabolism.

**Conclusions:**

A DNL gene activation profile in response to statins is associated with insulin resistance and the diabetic status of the patients. Identified molecular signatures thus suggest that statin treatment increases the risk for diabetes in humans at least in part via induction of DNL.

**Trial registration:**

NCT01129297. Registered May 242,010 (retrospectively registered).

**Electronic supplementary material:**

The online version of this article (10.1186/s12920-019-0536-1) contains supplementary material, which is available to authorized users.

## Background

Inhibitors of 3-hydroxy-3-methylglutaryl-coenzyme A (HMG-CoA) reductase (HMGCR, EC 1.1.1.88), statins, are widely used in the primary and secondary prevention of cardiovascular diseases, efficiently lowering serum low density lipoprotein cholesterol (LDL-C) levels in patients with dyslipidemia [1]. However, beside proven benefits some adverse effects of statin treatment has also been reported, including elevated liver enzymes, myopathies and neuropathies, limiting statin use in certain patient populations [2–4]. Moreover, in recent years, several randomized controlled trials (RCTs) [5–8] and observational studies [9–13] have reported an increased risk for new-onset type 2 diabetes mellitus (T2D) with statin treatment. Subsequent meta-analyses of results from primary and secondary prevention RCTs with various statins and dosages have confirmed the presence of excess risk for T2D with statin use [14, 15], the risk being enhanced by more intensive statin therapy [5, 6]. In RCTs, the point estimates for the associations have generally been in the range of 9–16% overall, and 24–28% for those with major T2D risk factors. Genetic polymorphisms with reduced HMGCR function are also associated with body weight gain, insulin resistance, and diabetes in humans [16]. This and other studies showing a relationship between HMGCR loss-of-function and higher risk for T2D support an on-target mechanism of the relationship [17]. However, the causative events for these associations are still unknown. Numerous mechanisms have been proposed for statin-associated diabetes risk, primarily related to increased insulin resistance and impaired insulin secretion. Animal models and in-vitro studies have shown that HMGCR inhibition has multiple down-stream effects that may increase diabetes risk. Statin-induced impairment of insulin signaling, adipocyte differentiation, pancreatic β-cell insulin secretion, mitochondrial dysfunction and other effects have been reported [18, 19]. Here, we explored the effects of statin treatment on human livers using a cohort of 910 severely obese patients with varying degrees of glucose tolerance and cardio-metabolic comorbidities such as hypertension, dyslipidemia and diabetes. Molecular signatures associated with statin treatment in human liver were identified by genome-wide analysis of gene expression patterns and subsequently correlated with anthropometric and metabolic parameters.

## Methods

### Patient cohort

The subjects enrolled in this study were participants of the Biological Atlas of Severe Obesity (ABOS) cohort (ClinicalTrials.gov identifier NCT01129297), an ongoing prospective cohort study for the longitudinal assessment of metabolic outcomes after weight loss surgery. Participants enrolled in the present study were of European (91.8%) and/or African (8.2%) ancestry. Biopsies were managed by the Lille University Hospital Biobank (CRB/CIC1403, brief registration number: BB0033–00030). The study design has been previously detailed [20, 21]. Briefly, all patients were severely obese adults who fulfilled the criteria for weight loss surgery, including severe obesity [body mass index (BMI) ≥40 kg/m^2^ or ≥ 35 kg/m^2^ with comorbidities] for at least 5 years and resistance to medical treatment, and the absence of medical or psychological contraindications to surgery. Patients with current excessive drinking (daily consumption of alcohol ≥20 g/day for women and ≥ 30 g/day for men), history of past excessive drinking for a period longer than 2 years at any time in the past 20 years, long-term consumption of hepatotoxic drugs, or positive screening for chronic liver diseases including positive testing for hepatitis B surface antigen and hepatitis C virus antibodies, evidence of genetic hemochromatosis, and age < 18 years were excluded. All enrolled patients gave their informed consent for a comprehensive metabolic phenotyping with tissue, plasma and serum sampling prior to the intervention. Hemoglobin A1c (HbA1c), fasting and 2 h blood glucose levels, fasting plasma levels of insulin and C-peptide, BMI and the homeostasis model assessment of insulin resistance (HOMA2-IR) were measured or calculated as described in the Additional file 1 section. Glucose tolerance and diabetes were defined using the guidelines of the American Diabetes Association. The diabetic status was attributed if patients exhibited a fasting blood glucose > 7.0 mmol/L and/or 2 h blood glucose > 11.1 mmol/L and/or HbA1c > 6.5 levels and/or were on antidiabetic treatment [22]. Hypertension was defined as systolic blood pressure (BP) ≥130 mmHg and/or diastolic BP > 80 mmHg and/or specific treatment. A total number of 173 (19.0%) out of the 910 patients included in this study were on statin medication with 69 (39.9%) being on atorvastatin, 48 (27.7%) on rosuvastatin, 36 (20.8%) on simvastatin, 19 (11.0%) on pravastatin and 1 (0.6%) on fluvastatin. Patient characteristics with key anthropometric and metabolic parameters, medications and comorbidities of the study cohort are presented in Table 1.

**Table 1:** Patient characteristics

Characteristics	n	Values
Age [yr]	910	41.7 ± 11.7
Females	910	658 (72.3%)
Diabetic father	860	198 (23.0%)
Diabetic mother	881	277 (31.4%)
Obese father	870	312 (35.9%)
Obese mother	889	451 (50.7%)
Body mass index [kg/m^2^]	910	46.7 ± 8.7
Waist circumference [cm]	891	130.3 ± 17.7
Systolic blood pressure [mmHg]	908	135.9 ± 18.6
Diastolic blood pressure [mmHg]	908	76.3 ± 14.2
Diabetes	910	350 (38.5%)
Patients on antidiabetic drugs	892	281 (31.5%)
Patients with number of antidiabetic drugs	892	
0		611 (68.5%)
1		126 (14.1%)
2		68 (7.6%)
> 2		87 (9.8%)
Patients on insulin treatment	910	89 (9.8%)
Fasting blood glucose [mmol/L]	898	5.6 (5.1 to 6.8)
Fasting plasma insulin [mUI/L]	897	14.1 (9.3 to 21.1)
Fasting plasma C-peptide [ng/mL]	701	3.8 (3.0 to 4.9)
2 h blood glucose [mmol/L]	868	7.5 (6.0 to 11.2)
2 h plasma insulin [mUI/L]	866	57.3 (30.1 to 102.3)
2 h plasma C-peptide [ng/mL]	618	10.5 (7.5 to 13.1)
HbA1c [%]	902	5.9 (5.5 to 6.6)
HOMA2-B	896	116.7 (82.4 to 162.8)
HOMA2-IR	896	2.2 (1.4 to 3.2)
Hypertension	910	727 (79.9%)
Patients on anti-hypertensive drugs	910	386 (42.4%)
Total cholesterol [mmol/L]	903	4.9 ± 1.0
LDL cholesterol [mmol/L]	896	3.0 ± 0.8
HDL cholesterol [mmol/L]	903	1.1 ± 0.3
Triglycerides [mmol/L]	903	1.7 ± 1.4
Dyslipidemia	910	904 (99.3%)
Patients on antidyslipidemia drugs	910	217 (23.8)
Patients on statins	910	173 (19.0)

Key anthropometric and metabolic parameters, medications and comorbidities of obese patients prior to surgery (*n* = number of patients with available data for the indicated parameter) are shown. Values are presented as numbers and percentage, mean ± SD or median (IQR) as appropriate. Abbreviations: *HbA1c* hemoglobin A1c, *HOMA2-B* homeostasis model assessment of β-cell function, *HOMA2-IR* homeostasis model assessment of insulin resistance, *LDL* low density lipoprotein, *HDL* high density lipoprotein, *IQR* interquartile range, *SD* standard deviation

### Measurement and calculation of clinical parameters

Plasma glucose levels were measured by the hexokinase method on an automatic analyzer (Roche Diagnostics, France). Plasma insulin was measured by immuno-reactive monoclonal assay using the Bi-Insulin kit (Cis-Bio International, France) with a sensitivity of 1 μUI/mL and an inter-assay coefficient of variation < 8%. Plasma C-peptide was measured by an immuno-metric assay run on a Cobas immunoanalyzer E601 (Roche Diagnostics, France) with a sensitivity of 0.01 ng/mL and an inter-assay coefficient of variation < 2.3%. The Homeostasis Model Assessment (HOMA) of insulin resistance and β-cell function indices HOMA2-IR and HOMA2-B were calculated using the HOMA2 calculator version 2.2.3 [23].

### Propensity score analysis

Propensity score-matched comparisons were performed to evaluate the difference in main diabetes and hepatic parameters between patients treated or not with statins. Quantitative variables are expressed as means (standard deviation) in the case of normal distribution or medians (interquartile range) otherwise. Categorical parameters are expressed as numbers (percentage). Normality of distributions was assessed using histograms and the Shapiro-Wilk test. The propensity score was estimated using a non-parsimonious multivariate logistic regression model, with statin treatment as the dependent variable and the following pre-specified factors as covariates: age, gender, parental familial history of diabetes, BMI, waist circumference, systolic and diastolic blood pressure, and use of antihypertensive drugs. Patients from the statin-users group were matched 1:1 to patients in the non-statin users group according to propensity score using the greedy nearest neighbor matching algorithm with a caliper width of 0.2 SD of logit of propensity score [24, 25]. To evaluate bias reduction using the propensity score matching method, the magnitude of the between-group differences was assessed by calculating absolute standardized differences (ASD), with an ASD > 10% indicated a meaningful imbalance in the baseline covariate [26]. Comparisons of main diabetes parameters between the statin and non-statin users matched groups were done using a generalized linear mixed model (GLMM) with binomial distribution and logit link function for binary parameters, a GLMM with multimodal distribution and cumulative logit link function for ordinal parameters, and linear mixed model for continuous parameters. To take into account the matched design, a random effect for matched sets was included into the GLMM and linear mixed models. To handle missing covariates values, multiple imputation procedure was used with a regression switching approach: chained equations with m = 10 imputations obtained using the R statistical software version 3.03 [27]. Imputation procedure was performed under the missing at random assumption using all variables listed in Table 1 (including treatment group) with a predictive mean matching method for continuous variables and multinomial or binary logistic regression model for categorical variables. In each imputed dataset, we calculated the propensity score, assembled a matched cohort, and estimated the effect size [28]. Therefore, we combined the effect size from each imputed dataset using Rubin’s rules [29]. Patient characteristics (other than diabetes and hepatic outcome parameters) were described according to statin treatment before and after propensity score matching (Additional file 1: Table S1).

### Microarray analysis

Total RNA from liver samples of 910 patients was extracted for Affymetrix microarray analysis from 30 mg frozen liver biopsies using Trizol reagent (Thermo Fisher Scientific) followed by a purification step on RNeasy columns (Qiagen). RNA purity and quantity was assessed using a Nanodrop spectrometer (Thermo Fisher Scientific). RNA integrity was quantified using the Agilent RNA6000 Nano assay and an Agilent 2100 BioAnalyzer. Nine hundred and ten samples complying with Affymetrix QC standards and a RNA integrity number (RIN) value of 5 to 7 were randomized for further processing and 300 ng RNA per sample was amplified using the Affymetrix WT amplification kit. Fragmented sscDNA was labelled using the Affymetrix WT Terminal Labeling Kit. Labeled DNA was then hybridized to Human Transcriptome Arrays 2.0 (Affymetrix) for 16 to 18 h at 45 °C and 60 rpm in a rotating hybridization oven (Hybridization Oven 640, Affymetrix). These high-resolution arrays contain > 6 million distinct oligonucleotide probes (25-mers) covering coding and non-coding transcripts, enabling the identification and analysis of differential expression at the gene and exon level. After washing, arrays were scanned with the GeneChip Scanner 3000 7G (Affymetrix), controlled by the Affymetrix software GeneChip Operating System (GCOS) v1.4. Quality controls (pm_mean / background_mean, pos_vs_neg_auc, all_probeset_mad_residual_mean), hybridization controls (bioB, bioC, bioD and cre) and polyA controls (lys, phe, thr and dap) were performed according to the Affymetrix quality criteria using the Expression Console software (Affymetrix). The HUGO Gene Nomenclature Committee (http://www.genenames.org) gene names were used in this study. Affymetrix raw files are available at GEO under the accession number GSE130991.

### Quantitative reverse transcription-polymerase chain reaction analysis

Ten statin-regulated genes identified by the microarray analysis corresponding to cholesterogenic and lipogenic pathways were selected. Randomly selected RNAs corresponding to 40 statin-treated and 40 non-statin treated patients were analyzed by RT-qPCR. Reverse transcription was performed using random hexamers as recommended by the manufacturer (Promega). The cDNAs were analyzed using TaqMan PCR master mix (Thermo Fisher Scientific) and a mix of 18S and gene-specific primer mix. The 18S primers and primer specific mix for *TM7SF2*, *FDPS*, *LSS*, *SQLE*, *HMGCR*, *ELOVL6*, *FADS2*, *SCD*, *ACACA*, and *FASN* (Hs00162807_m1, Hs00266635_m1, Hs01552329_m1, Hs01123768_m1, Hs00168352_m1, Hs00907564_m1, Hs00927433_m1, Hs01682761_m1, Hs01046047_m1, Hs00188012_m1 respectively) were from Applied Biosystems / Thermo Fischer Scientific. Reaction. PCR analysis was carried out with an ABI Prism 7700 (Perkin Elmer, France).

### Bioinformatics analysis

Microarray data analysis was performed using the ArrayStudio software package, version 10.0.1.75 (Omicsoft). Raw data from Affymetrix microarrays were first processed with robust multi-array average (RMA) as a normalization method and then log_2_-transformed. Affymetrix probe set identifiers with intensity signals of < 4 in at least 25% of the sample groups were filtered out to exclude minimally expressed genes from the analysis. Principal component analysis (PCA) was applied to all samples as a quality control assessment. To detect differentially expressed genes, a pairwise ANOVA statistical test was applied to the comparison between 157 statin-treated and 157 non-statin treated patients of the matched cohort (obtained in the first imputed dataset). *P* values were adjusted using the Benjamini-Hochberg procedure to control false discovery rates. Criteria for determining differentially expressed genes with statistical significance were changes in expression levels with an adjusted P value < 0.05. Data sets containing Affymetrix probe set identifiers and corresponding statistical values were uploaded into the Ingenuity Pathway Analysis (IPA) software application, version 3,605,602 (Qiagen) and each identifier was mapped to a gene using the IPA library. Pathway analysis identified canonical pathways from the IPA library that were most significantly related to the data set. The significance of the association between the data set and the canonical pathways was measured in two ways: (1) the ratio of the number of molecules from the data set that map to the pathway divided by the total number of molecules that map to the canonical pathway, (2) the Fisher’s exact test was used to calculate a *P* value determining the probability that the association between the genes in the data set and the canonical pathway is explained by chance alone. The IPA downstream effects analysis was used to identify the biological functions and disease processes that were most significantly related to the data set. Right-tailed Fisher’s exact test was used to calculate a P value determining the probability that each biological function and disease assigned to these data sets are due to chance alone. Downstream effects analysis was used to predict increases or decreases of these biological functions occurring in liver samples after statin treatment by integrating the direction change of the differentially expressed genes into a z-score algorithm calculation. The same algorithm has been used in up-stream effector analysis to predict activation or inhibition of regulators. The Pearson correlation analysis between gene expression levels and clinical parameters was applied to all genes from the microarray. Differences in the diabetes status within the statin-treated and non-statin treated patient group of the matched cohort were adjusted using a general linear model.

## Results

### Cohort characteristics

A total number of 910 obese patients consecutively recruited for body weight loss surgery were included. Table 1 shows baseline patient characteristics with key anthropometric and metabolic parameters, medications and comorbidities of obese patients prior to surgery. The mean patient age was 41.7 (±11.7), 658 (72.3%) patients were women and the mean BMI was 46.7 (±8.7). Three hundred and fifty patients were diabetics (38.5%) amongst whom 89 were treated with insulin. Patient characteristics (other than main diabetes parameters) according to statin treatment before and after propensity score-matching are reported in Additional file 1: Table S1. Before matching, several significant differences (absolute standardized difference > 20%) were found. Statin users were older, predominantly male, had a larger waist circumference and hypertension was more prevalent than in non-statin users.

### Propensity score matching reveals an increased prevalence of diabetes in statin users

Propensity score matching for these pre-specified confounding factors reduced these differences with all absolute standardized difference < 10%, thus resulting in 2 well-balanced study groups with 157 patients each after matching (mean number of imputated datasets). As expected, lipid profiles differed between the two study groups, with lower total cholesterol and LDL-C levels found in statin users. Table 2 shows the main diabetes parameters in the statin and non-statin group after propensity score-matching. In this matched patient cohort, the prevalence of diabetes in statin users (75.1%) was significantly higher than in non-statin users (53.0%; OR = 2.67; 95%CI, 1.60–4.45; *P* = 0.0002). A similar difference was observed when considering the use of antidiabetic drugs or the number of antidiabetic drugs. Significantly higher levels in fasting blood glucose, 2-h blood glucose and HbA1c were also found in statin users compared to non-statin users (Table 2).

**Table 2:** Propensity-score matching of statin-treated with non statin-treated patients

	Statin group (*n* = 157)	Non-statin group (*n* = 157)	Effect size	Values (95% CI)	*P* value
Diabetes	117 (75.1)	83 (53.0)	Odds ratio	2.67 (1.60 to 4.45)	0.0002
Patients on antidiabetic drugs	110 (70.5)	67 (42.9)	Odds ratio	3.18 (1.96 to 5.15)	< 0.0001
Patients with number of antidiabetic drugs					
0	46 (29.5)	89 (57.1)	Common odds ratio	2.88 (1.83 to 4.53)	< 0.0001
1	39 (24.7)	27 (17.0)			
2	26 (17.0)	18 (11.6)			
> 2	45 (28.8)	22 (14.3)			
Patients on insulin treatment	42 (26.8)	23 (14.4)	Odds ratio	2.17 (1.25 to 3.74)	0.006
Fasting blood glucose [mmol/L]	7.1 (5.9 to 10.1)	6.0 (5.4 to 8.0)	Mean difference^a^	0.14 (0.06 to 0.22)	0.0002
2 h blood glucose [mmol/L]	11.6 (7.4 to 17.0)	9.0 (6.6 to 13.8)	Mean difference^a^	0.18 (0.06 to 0.29)	0.002
HbA1c [%]	6.8 (6.0 to 8.2)	6.2 (5.7 to 7.1)	Mean difference^a^	0.09 (0.04 to 0.14)	0.0001

The comparison of the main patient characteristics between statin and non-statin treatment groups after propensity score-matching and multiple imputation is shown. Values are presented as n (%) or median (IQR) unless otherwise indicated. ^a^calculated on log-transformed data. Abbreviations: *HbA1c* hemoglobin A1c

### Statin treatment in humans alter the cholesterol biosynthetic pathway

High-quality transcriptomics data were obtained from 910 human liver tissue samples from the ABOS cohort. No clear separation was observed between samples from statin and non-statin treated patients by a principal component analysis (PCA), indicative of technical homogeneity and of discrete gene expression differences (Additional file 1: Figure S1). Differential expression analysis using a pairwise ANOVA test led to the identification of 135 out of 70,523 Affymetrix transcript identifiers which were statistically significantly regulated by statin treatment in the matched cohort. These 135 transcripts were mapped to 98 non-redundant, protein-coding genes with functional annotations (Additional file 1: Figure S2 and Table S2) and displayed log_2_ fold-change (FC) ranging from − 1.3 to 2.0 with adjusted *P* values < 0.05. More genes were found to be upregulated in the statin-treated group (up: *n* = 85 vs down: *n* = 13). The gene with the most statistically significant up-regulation in response to statins encodes farnesyl-diphosphate synthase (*FDPS*: log_2_FC = 1.7, *P* = 4.3 × 10^− 23^), which catalyzes the production of farnesyl-pyrophosphate, a key intermediate in cholesterol and sterol biosynthesis (Additional file 1: Figure S3). The gene displaying the highest FC was 3-hydroxy-3-methylglutaryl-coenzyme A synthase (*HMGCS1*, log_2_FC = 2, *P* = 9.7 × 10^− 19^), which catalyzes the first step of the cholesterol biosynthetic pathway to generate HMG-CoA from acetyl-CoA. As expected, the gene encoding for the primary target of statins, 3-hydroxy-3-methylglutaryl-coenzyme A reductase (*HMGCR*: log_2_FC = 1.7, *P* = 1.1 × 10^− 14^) was upregulated in statin-treated patients due to the well-known counter-regulation effect mediated by sterol regulatory element-binding protein-2 (SREBP2) (Additional file 1: Figure S3). Hepatic low density lipoprotein receptor (*LDLR*: log_2_FC = 1.2, *P* = 2.0 × 10^− 04^) mRNA was also significantly higher in statin-treated patients, a well-established adaptative mechanism through which statins promote uptake of LDL particles from the blood by the liver to maintain cholesterol homeostasis (Additional file 1: Figure S3). The differential expression of statin-regulated genes from the cholesterol biosynthetic pathway could be confirmed after adjustment for differences in the diabetes status within the statin-treated and non-statin treated patient group (Additional file 1: Table S2B). No significant differences in liver transcriptomes could be observed between the different statin drugs prescribed in this cohort (data not shown). The differential expression of 8 out of 10 selected statin-regulated genes identified by microarray analysis could be confirmed by quantitative reverse transcription polymerase chain reaction (RT-qPCR) assays (Additional file 1: Table S3).

### Pathway enrichment analysis identifies the SREBP pathway as sensitive to statin treatment

**Fig. 1:**
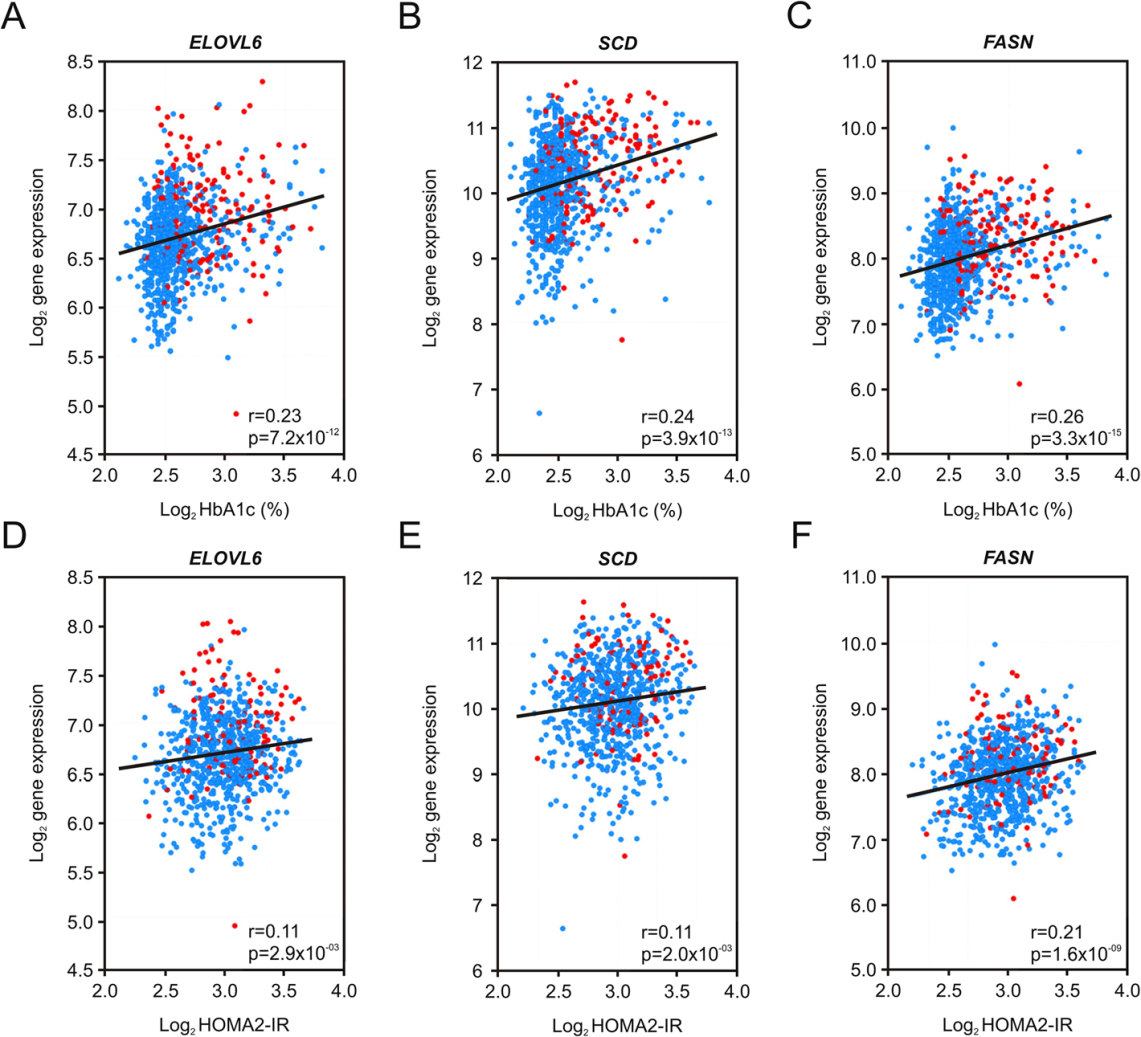
*Correlation between gene expression, glucose and insulin homeostasis.* Pearson correlation plots between log_2_ gene expression values of key lipogenic genes and HbA1c (**a**-**c**) or HOMA2-IR (**d**-**f**) are shown. Genes are fatty acid elongase-6 (*ELOVL6*), stearoyl-CoA desaturase (*SCD*) and fatty acid synthase (*FASN*). Liver samples from non-statin treated patients (*n* = 747) are indicated as blue dots, liver samples from statin-treated patients (*n* = 173) are indicated as red dots. The following correlation coefficients and *P* values were calculated for HbA1c: *ELOVL6*: r = 0.23, *P* = 7.2 × 10^− 12^; *SCD*: r = 0.24, *P* = 3.9 × 10^− 13^; *FASN*: r = 0.26, *P* = 3.3 × 10^− 15^. The following correlation coefficients and P values were calculated for HOMA2-IR: *ELOVL6*: r = 0.11, *P* = 2.9 × 10^− 03^; *SCD*: r = 0.11, *P* = 2.0 × 10^− 03^; *FASN*: r = 0.21, *P* = 1.6 × 10^− 09^. Abbreviations: HbA1c: hemoglobin A1c, HOMA2-IR: homeostasis model assessment of insulin resistance

**Fig. 2:**
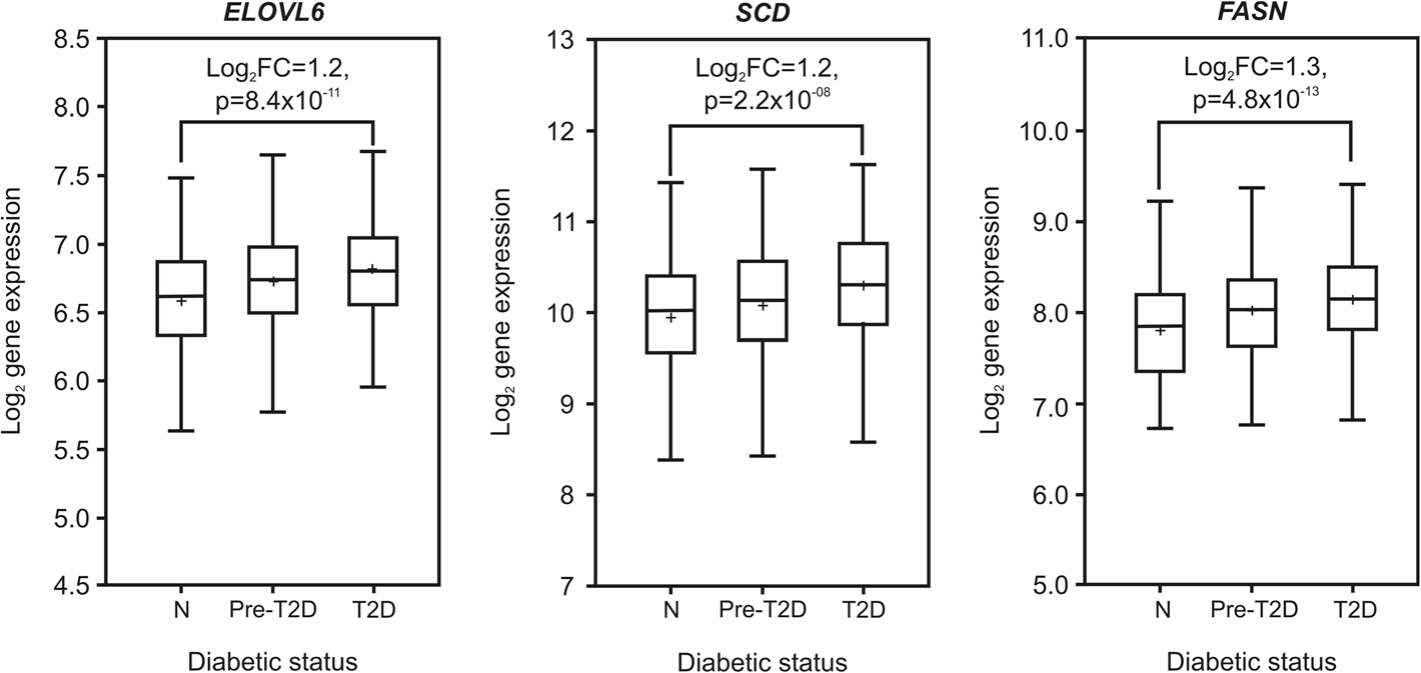
*Gene expression values in healthy, pre-diabetic and type 2 diabetics patients.* Boxplots show log_2_ gene expression values of key lipogenic genes according to the diabetic status. N: normal patients (*n* = 241), P: pre-diabetic patients (*n* = 319) and D: diabetic patients (*n* = 350). Genes are fatty acid elongase-6 (*ELOVL6*), stearoyl-CoA desaturase (*SCD*) and fatty acid synthase (*FASN*). The following *P* values and log_2_ fold changes (log_2_FC) were calculated from pairwise comparisons of diabetic vs normal patients: *ELOVL6*: log_2_FC = 1.2, *P* = 8.4 × 10^− 11^; *SCD*: log_2_FC = 1.2, *P* = 2.2 × 10^− 08^ and *FASN*: log_2_FC = 1.3, *P* = 4.8 × 10^− 13^

To uncover key pathways associated with statin treatment, data analysis was performed with the Ingenuity software application. Table 3 shows enriched metabolic and signaling pathways, disease processes or upstream regulators identified from the list of differentially expressed genes in response to statin treatment in the matched patient cohort. The main enriched pathways were cholesterol biosynthesis (*P* = 1.2 × 10^− 40^), fatty acid metabolism (*P* = 2.5 × 10^− 09^), Liver X Receptor/Retinoid X receptor (LXR/RXR) signaling (*P* = 2.3 × 10^− 08^) and AMP-activated protein kinase (AMPK) signaling (*P* = 9.9 × 10^− 03^). From the signaling pathways, AMPK signaling was predicted to be decreased by statin treatment (Z-score = − 1.26), whereas LXR/RXR signaling was predicted to be increased (Z-score = 1.43). Transcriptional changes in the AMPK and LXR/RXR signaling pathways may at least partially explain identified metabolic disease processes such as insulin resistance (*P* = 6.9 × 10^− 06^) and type 2 diabetes mellitus (*P* = 1.3 × 10^− 03^), which are all significantly modulated in statin-treated patients. Upstream effector analysis led to the identification of main transcription factors known to control the majority of transcriptional changes in both cholesterol and fatty acid metabolic pathways. Sterol regulatory element-binding protein-1 (*SREBP1*, Z-score = 4.64), Sterol regulatory element-binding protein-2 (*SREBP2*, Z-score = 4.74), and SREBP-cleavage activating protein (*SCAP*, Z-score = 4.84) were predicted to be upregulated, whereas insulin induced gene-1, an inhibitor of SCAP and activator of HMGCR proteasomal degradation (*INSIG1*, Z-score = − 3.77), was predicted to be inhibited by statins. In total, 44 out of 98 statin-regulated genes were directly involved in cholesterol, fatty acid and triglyceride metabolism. A hierarchical model illustrating these findings shows the role of the upstream regulators SREBP1, SREBP2, SCAP and INSIG1 (Additional file 1: Figure S4). Target genes for SREBP1 AND SREBP2 include key lipogenic genes such as acetyl-CoA carboxylase-alpha (*ACACA*), fatty acid synthase (*FASN*), fatty acid elongase-6 (*ELOVL6*) and stearoyl-CoA desaturase (*SCD*), involved in fatty acid (FA) biosynthesis from acetyl-coA, FA elongation and desaturation, all of them having been linked to the progression of insulin resistance and diabetes [30–33]. Therefore, we explored a potential correlation between expression levels of these genes with parameters of glucose homeostasis and insulin resistance. As a result, positive correlations were found for HbA1c, fasting blood glucose, 2 h blood glucose and HOMA2-IR with *P* values varying from 2.9 × 10^− 03^ to 3.3 × 10^− 15^ for *ELOVL6*, *SCD* and *FASN* (Fig. 1, data not shown for fasting and 2 h blood glucose). We further analyzed the expression level of these lipogenic genes as a function of the diabetic status of the patients. Patients were classified into three different groups: normal, pre-diabetic and diabetic. Highest expression values were always found in diabetic patients and lowest values in normal patients with P values varying from 2.2 × 10^− 08^ to 4.8 × 10^− 13^ for *ACACA*, *SCD* and *FASN* (Fig. 2).

**Table 3:** Pathway enrichment analysis of statin-dysregulated genes

Pathway (1) or Disease (2) or Regulator (3)	*P* value	Prediction	Activation Z-score	# Genes
1. Superpathway of cholesterol biosynthesis	1.2 × 10^−40^	increased	4.36	19
1. Fatty acid metabolism	2.5 × 10^−09^	increased	1.82	18
1. LXR/RXR signaling	2.3 × 10^−08^	increased	1.43	8
1. AMPK signaling	9.9 × 10^−03^	decreased	−1.26	4
2. Metabolic disease	9.6 × 10^− 08^		n.a.	31
2. Insulin resistance	6.9 × 10^−06^		n.a.	10
2. Type II diabetes mellitus	1.3 × 10^−03^		n.a.	15
3. SREBF1	1.5 × 10^−34^	activated	4.64	29
3. SREBF2	1.3 × 10^−56^	activated	4.74	31
3. SCAP	3.4 × 10^−52^	activated	4.84	27
3. INSIG1	1.3 × 10^−40^	inhibited	−3.77	26

Main enriched metabolic and signaling pathways (1), disease processes (2) and regulators (3) modulated by statin treatment in the propensity score-matched patient cohort (*n* = 314) are shown. The analysis was performed using the Ingenuity Pathway Analysis (IPA) using a list of 98 statin-regulated genes (see Additional file 1: Table S2). The total number of statin-regulated genes assigned to each pathway or disease process is given under the “# genes” column. For regulators (3), the number of downstream target genes is given (all corresponding gene symbols are listed in Additional file 1: Table S4). The Z-score indicates the match between observed and predicted up- or downregulation patterns. Abbreviations: *LXR/RXR* liver X receptor/retinoid X receptor, *AMPK* AMP-activated protein kinase, *SREBF* sterol regulatory element-binding factor, *SCAP* SREBP-cleavage activating protein, *INSIG1* insulin induced gene-1, *n.a.* no molecular activity prediction available

## Discussion

In this study, we investigated the association of statin treatment with glucose homeostasis by analyzing genome-wide transcriptomic profiles from liver tissue samples from a cohort of 910 subjects displaying varying degrees of obesity and glucose tolerance. After propensity score-matching of all patients with regard to confounding parameters such as age, gender, BMI and cardiovascular risk, we confirmed the higher prevalence of diabetes and impairment of glucose homeostasis in statin-treated patients as previously reported [5, 6, 8–15]. To identify liver-related molecular mechanisms potentially explaining statin-associated diabetes in the matched cohort, transcriptomic profiles correlated to clinico-biological phenotypes were assigned to biological pathways or networks. Molecular signatures of statin treatment on hepatic gene expression have only been investigated in vitro or in animal studies so far. In human primary hepatocytes, statins upregulate genes involved in cholesterol metabolism, glucose and fatty acid homeostasis, suggesting a role of these genes in higher blood glucose levels observed in statin-treated patients [34]. Increased de novo lipogenesis (DNL) and liver fat accumulation has been found after statin treatment in Zucker rats (fa/fa), a genetic model for obesity, dyslipidemia and insulin resistance [35]. In our study, we could identify a total number of 44 statin-regulated genes, which are mainly part of the cholesterogenic (e.g. *HMGCR*, *HMGCS1*, *FDPS*, *LSS* and *SQLE*) and lipogenic (e.g. *ACACA*, *SCD*, *FASN*, *FADS1* and *ELOVL6*) pathways. A mechanistic hypothesis for this observation may rely on the identification of SREBP1, SREBP2, SCAP and INSIG1 as highest-scoring upstream regulators which are activated or inhibited by statin treatment. SREBP1 is responsible for regulating genes required for *DNL*, whereas SREBP2 regulates genes of cholesterol metabolism [36]. Activation of the SREBP transcription factors requires simultaneous inhibition of INSIG1 and activation of SCAP. In the presence of low cholesterol, SCAP binds to SREBPs and mediates their transport from the endoplasmic reticulum (ER) to the Golgi. After proteolytic activation, SREBPs then enter the nucleus and initiate transcription of their downstream targets. In the presence of high cholesterol, INSIG1 prevents the SCAP-SREBP complex to exit the endoplasmic reticulum (ER), thereby blocking transcriptional activity [36, 37]. Regulation of SREBPs occurs at the level of SREBP synthesis, proteolytic activation, transcriptional activity, and proteasome-dependent degradation by integrating multiple metabolite signals (e.g. sterols) and pathways (e.g. insulin signaling, LXR and AMPK signaling) [37]. The expression of 8 statin-regulated genes identified from our study were mapped to the hepatic LXR/RXR signaling pathway, including genes from cholesterol biosynthesis (e.g. *HMGCR*, *CYP51A1*), cholesterol transport (*ABCG1*) and lipogenesis (*ACACA*, *FASN*, *SCD*). The liver X receptor (LXR) is sensitive to oxysterol derivatives, forms a heterodimer with the retinoid X receptor (RXR) and regulates the expression of target genes linked to lipid homeostasis by binding to LXR response elements [38]. In liver, LXR activation also stimulates the expression of SREBP1 leading to increased lipogenesis and lipid accumulation [38]. Increased LXR/RXR and decreased AMPK signaling induced by statins seems to be highly connected to SREBP activation and transcription of downstream target genes identified from our study. In the cohort presented here, liver expression levels from statin-regulated lipogenic genes such as *ELOVL6*, *SCD* and *FASN,* which are under transcriptional control by SREBP1, were positively correlated with insulin resistance and the diabetic status. Indeed, many genes identified in statin-treated patients are directly linked to insulin resistance and diabetes and play defined roles in hepatic insulin receptor, AMPK and LXR/RXR signaling, clearly pointing to diabetogenic effects of statins. Statin-induced DNL and accumulation of free fatty acids and triacylglycerol metabolites in liver (e.g. fatty acyl-CoA, diacylglycerol, ceramides) may also trigger oxidative stress and mitochondrial dysfunction, thereby contributing to insulin resistance and diabetes [39].

The non-interventional, cross-sectional design of our study is an important limitation. Statin-treated patients in the cohort were indeed older and had an overall higher incidence rate of cardiovascular and metabolic diseases. However, the large number of patients enrolled allowed us to confirm the statistical association between statin treatment and abnormal glucose parameters after adjustment for multiple confounders. Intensity of statin therapy has been reported to affect diabetes risk and might differ between patients of our study [6]. No conclusions could be made about this.

Genome-wide transcriptomic profiling of liver tissue samples from a large cohort of severely obese patients provides a novel global insight into hepatic effects of statins and demonstrates that statin treatment in human is associated with hepatic DNL. Furthermore, our results favor a class effect since no significant differences between molecular signatures from five different statin drugs could be identified. Pathway analysis of our data indicates that DNL in response to statins is mediated by the activation of SREBP1 and a concomitant upregulation of key genes from fatty acid and triglyceride metabolism.

## Conclusions

Our data indicate that DNL in response to statins is significantly associated with insulin resistance and the diabetic status of the patients. Decreased hepatic AMPK signaling and increased LXR/RXR signaling might be a possible explanation, but additional studies are needed to further explore other potential mechanisms for statin-associated diabetes. We suggest that patients at high risk for type 2 diabetes should be carefully monitored when statin therapy is prescribed. If necessary, other lipid-lowering therapies than HMG-CoA reductase inhibition may be considered.

## Additional file

Additional file 1: **Figure S1.** Principal component analysis (PCA) from 910 liver biopsies. **Figure S2.** Differential gene expression in the propensity score-matched cohort . **Figure S3.** Gene expression values of representative genes. **Figure S4.** Gene network in statin-treated patients. **Table S1.** Patient characteristics in the statin and non-statin treatment groups before and after propensity score-matching. **Table S2.** A. Differentially expressed genes between statin and non-statin treated patient groups from the matched patient cohort with Pearson correlation parameters to HbA1c and HOMA2-IR. B: Top 10 differentially expressed genes between statin and non-statin treated patient groups from the matched patient cohort with and without adjustment for diabetes status. **Table S3.** Q-PCR validation of 10 statin-regulated genes identified from the Affymetrix microarray analysis. **Table S4.** Pathway assignment to statin-dysregulated genes. (DOCX 806 kb)

## Data Availability

The datasets used and/or analyzed during the current study are available from GEO (GSE130991).

## Abbreviations

*ABCG1*: ATP binding cassette subfamily G member 1
*ACACA*: acetyl-CoA carboxylase alpha
AMPK: AMP-activated protein kinase
ASD: Absolute standardized differences
BMI: Body mass index
BP: Systolic blood pressure
CI: Confidence interval
*CYP51A1*: Cytochrome P450 family 51 subfamily A member 1
DNL: De novo lipogenesis
*ELOVL6*: Fatty acid elongase 6
ER: Endoplasmic reticulum
*FADS1*: Fatty acid desaturase 1
*FASN*: Fatty acid synthase
*FDPS*: Farnesyl diphosphate synthase
GLMM: Generalized linear mixed model
HbA1c: Hemoglobin A1c
HMG-CoA: 3-hydroxy-3-methylglutaryl-coenzyme A
HMGCR: 3-hydroxy-3-methylglutaryl-coenzyme A reductase
*HMGCS1*: 3-Hydroxy-3-Methylglutaryl-CoA Synthase 1
HOMA2-IR: Homeostasis model assessment of insulin resistance
*INSIG1*: Insulin induced gene-1
IPA: Ingenuity Pathway Analysis
LDL-C: Low density lipoprotein cholesterol
*LDLR*: Low density lipoprotein receptor
*LSS*: Lanosterol synthase
LXR: Liver X Receptor
OR: Odds ratio
PCA: Principal component analysis
RCTs: Randomized controlled trials
RMA: Robust multi-array average
RT-qPCR: Reverse transcription-quantitative polymerase chain reaction
RXR: Retinoid X receptor
*SCAP*: SREBP-cleavage activating protein
*SCD*: Stearoyl-CoA desaturase
*SQLE*: Squalene epoxidase
SREBP1: Sterol regulatory element-binding protein 1
SREBP2: Sterol regulatory element-binding protein-2
T2D: Type 2 diabetes mellitus
