# Supplementary material for: Hepatic transcriptomic signatures of statin treatment are associated with impaired glucose homeostasis in severely obese patients
Source: BMC Med Genomics. 2019 Jun 3;12:80. doi: 10.1186/s12920-019-0536-1 (PMC6545676; doi:10.1186/s12920-019-0536-1)
Supplement: Supplementary file 1 — Figure S1. Principal component analysis (PCA) from 910 liver biopsies. Figure S2. Differential gene expression in the propensity score-matched cohort . Figure S3. Gene expression values of representative genes. Figure S4. Gene network in statin-treated patients. Table S1. Patient characteristics in the statin and non-statin treatment groups before and after propensity score-matching. Table S2. A. Differentially expressed genes between statin and non-statin treated patient groups from the matched patient cohort with Pearson correlation parameters to HbA1c and HOMA2-IR. B: Top 10 differentially expressed genes between statin and non-statin treated patient groups from the matched patient cohort with and without adjustment for diabetes status. Table S3. Q-PCR validation of 10 statin-regulated genes identified from the Affymetrix microarray analysis. Table S4. Pathway assignment to statin-dysregulated genes. (DOCX 806 kb) [file 12920_2019_536_MOESM1_ESM.docx]

**Additional file 1**

Hepatic molecular signatures of statin treatment are associated with impaired glucose homeostasis in severely obese patients

Daniel Margerie^1 *^, Philippe Lefebvre^2 *^, Violeta Raverdy^3,5^, Uwe Schwahn^1^, Hartmut Ruetten^1^, Philip Larsen^1^, Alain Duhamel^4^, Julien Labreuche^4^, Dorothée Thuillier^3^, Bruno Derudas^2^, Céline Gheeraert^2^, Hélène Dehondt^2^, Quentin Dhalluin^2^, Jérémy Alexandre^2^, Robert Caiazzo^3,5^, Pamela Nesslany^5^, Helene Verkindt^5^, Francois Pattou^3,5^ *, and Bart Staels^2^ .

^1^ Sanofi Aventis Deutschland GmbH, Research & Development, D-65926 Frankfurt, Germany

^2^ Univ. Lille, INSERM, CHU Lille, Institut Pasteur de Lille, U1011 – EGID, F-59000 Lille, France

^3^ Univ. Lille, Inserm, CHU Lille, Institut Pasteur de Lille, U1011 – EGID, F-59000 Lille, France

^4^ CHU Lille, Department of Biostatistics, F-59000 Lille, France

^5^ CHU Lille, Department of General and Endocrine Surgery, F-59000 Lille, France

**
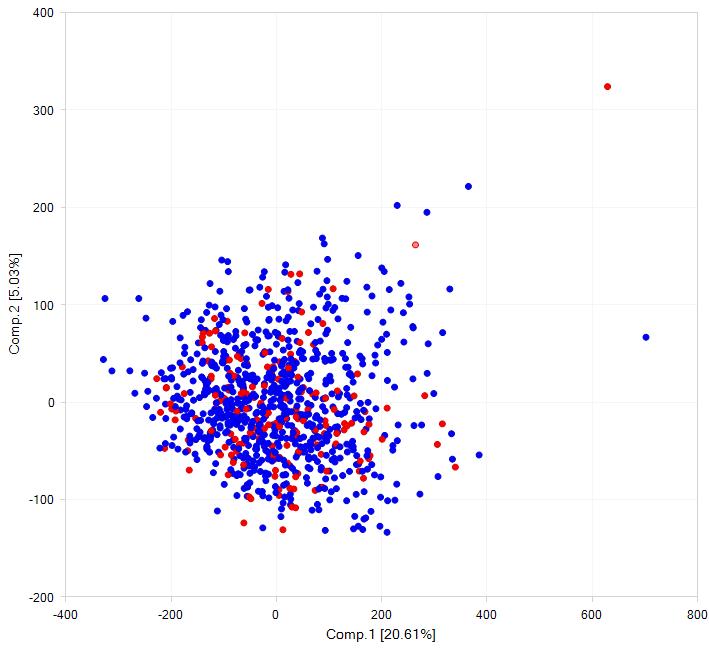
Figure S1**: ***Principal component analysis (PCA) from 910 liver biopsies.*** The PCA is based on all 70,523 transcript IDs from the Affymetrix Human Transcriptome Array (HTA) 2.0. Data points marked in red represent liver samples from statin-treated patients (n=173), data points marked in blue represent samples from non-statin treated patients (n=737).

**
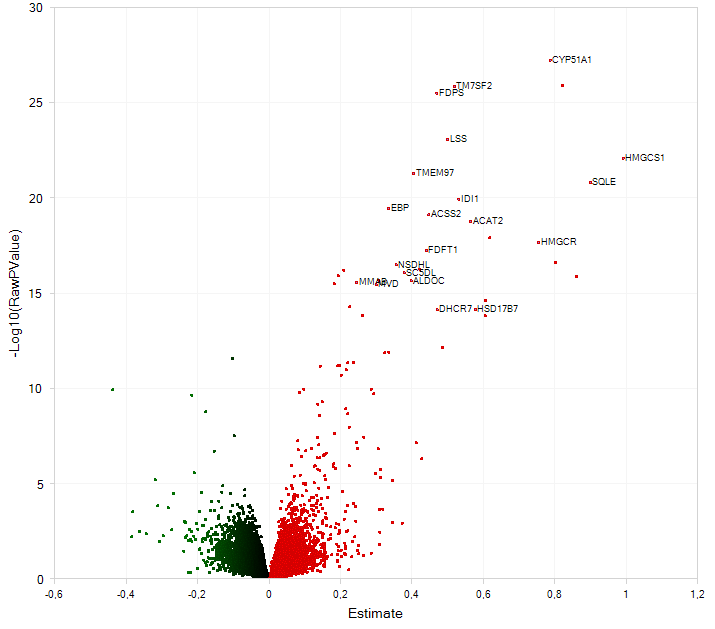
****Figure S2:** ***Differential gene expression in the propensity score-matched cohort*** . The volcano-plot is based on the pairwise comparison of statin-treated (n=157) vs non-statin-treated (n=157) patients of the matched patient cohort and shows the minus (-) log_10_ adjusted P value on the Y-axis vs the fold-change estimate on the X-axis. The estimate is defined as the statistically adjusted difference between the means of the two compared groups (ANOVA). Top 20 differentially expressed genes with adjusted P values < 0.05 and log_2_ fold-changes (FC) >1.2 are indicated (more details about these genes are given in Supplemental Table 2). Differentially expressed genes in green: downregulated by statins, red: upregulated by statins.


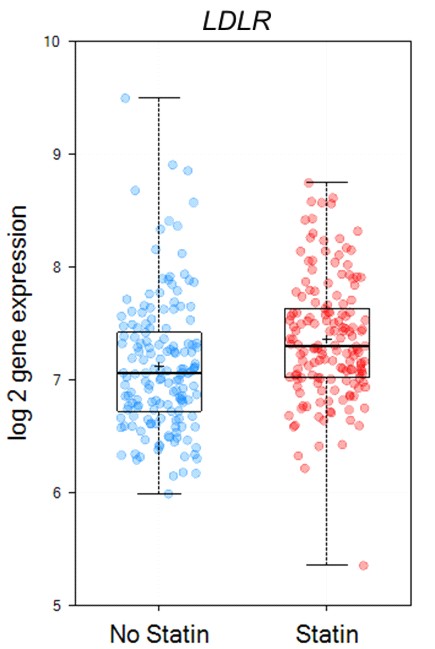

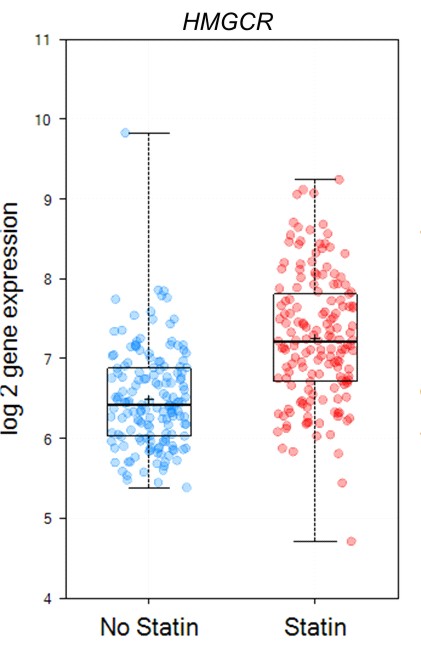

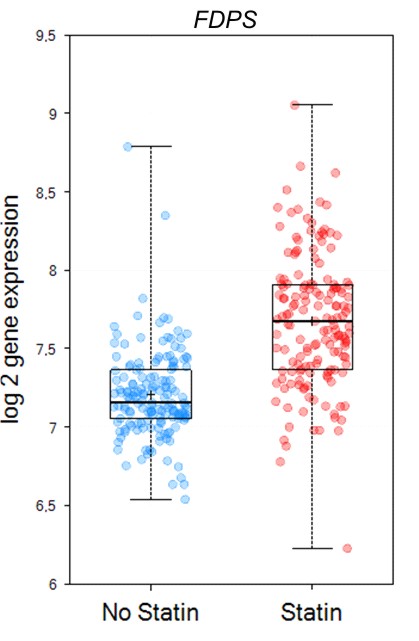


**Figure S3:** ***Gene expression values of representative genes.*** Boxplots with log_2_ gene expression values of key metabolic genes in liver samples from the matched patient cohort are shown according to statin treatment. Genes are farnesyl-diphosphate synthase (*FDPS*: log_2_FC=1.7, P=4.3x10^-23^), 3-hydroxy-3-methylglutaryl-CoA reductase (*HMGCR*: log_2_FC=1.7, P=1.1x10^-14^) and low density lipoprotein receptor (*LDLR*: log_2_FC=1.2, P=2.0x10^-04^). Liver samples from statin-treated group are marked red (n=157) and liver samples from the non-statin treated group are marked blue (n=157). Abbreviations: FC:fold change.


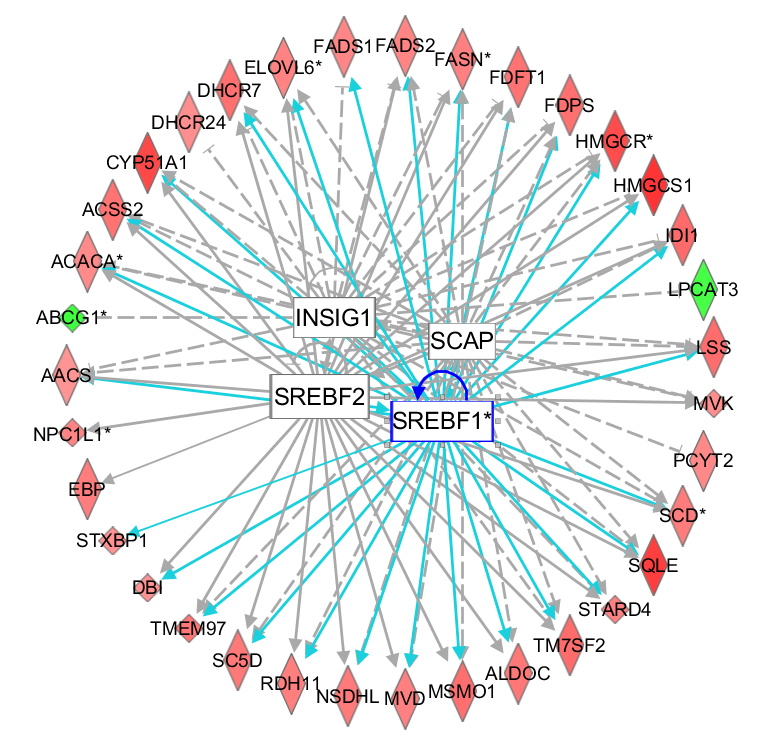


**Figure S4:** ***Gene network in statin-treated patients.*** The network illustrates statin-induced transcriptional upregulation (red) and downregulation (green) of 39 genes involved in cholesterol (e.g. *HMGCR*, *HMGCS1*, *SQLE*), fatty acid and triglyceride metabolism (e.g. *ACACA*, *FASN*, *SCD*) in the matched patient cohort, mediated by the predicted inhibition of INSIG1, activation of SCAP and subsequent activation of SREBP1 and SREBP2, encoded by the *SREBF1* and *SREBF2* genes. Lipid genes activated by SREBP1 are highlighted by interactions marked in blue. Genes marked with * are linked to hepatic steatosis according to literature findings reported by IPA.

| **Characteristics** | **Before Propensity Score-Matching*** | | | **After Propensity Score-Matching*** | | |
| --- | --- | --- | --- | --- | --- | --- |
|  | **Statin group (n=173)** | **Non-statin group (n=737)** | **ASD, (%)** | **Statin group (n=157)** | **Non-statin group (n=157)** | **ASD, (%)** |
| Age† [yr] | 52.1 ± 7.4 | 39.2 ± 11.1 | 136.1 | 51.4 ± 7.3 | 51.2 ± 8.3 | 2.6 |
| Females† | 96 (55.5) | 562 (76.3) | 44.9 | 90 (57.5) | 96 (61.6) | 8.2 |
| Diabetic father† | 41 (23.4) | 170 (23.1) | 0.7 | 37 (23.4) | 36 (23.1) | 0.8 |
| Diabetic mother† | 67 (38.7) | 223 (30.2) | 18.0 | 57 (36.7) | 62 (39.9) | 6.6 |
| Body mass index†* [kg/m^2^] | 46.1 ± 7.2 | 46.9 ± 9.0 | 9.7 | 46.2 ± 7.0 | 46.7 ± 9.2 | 6.3 |
| Waist circumference† [cm] | 134.8 ± 16.0 | 129.2 ± 18.3 | 32.2 | 134.3 ± 15.1 | 134.9 ± 20.2 | 4.3 |
| Systolic BP† [mmHg] | 137.4 ± 18.0 | 135.6 ± 18.8 | 10.0 | 138.0 ± 18.4 | 137.7 ± 22.6 | 2.2 |
| Diastolic BP† [mmHg] | 76.0 ± 12.9 | 76.4 ± 14.5 | 2.7 | 76.7 ± 13.1 | 76.1 ± 16.8 | 4.6 |
| Fasting plasma insulin [mUI/L] | 17.7 (11.0 to 31.6) | 13.8 (9.0 to 19.8) | 42.0 | 18.4 (11.1 to 31.5) | 15.5 (10.0 to 24.9) | 22.6 |
| Fasting plasma C-peptide [ng/ml] | 4.0 (3.0 to 5.3) | 3.8 (3.0 to 4.8) | 12.7 | 4.1 (3.1 to 5.4) | 4.1 (3.2 to 5.4) | 2.6 |
| 2 hour plasma insulin [mUI/L] | 50.9 (28.4 to 103.2) | 58.0 (29.9 to 99.9) | 2.3 | 51.6 (27.9 to 99.4) | 59.8 (33.1 to 104.7) | 5.5 |
| 2 hour plasma C-peptide [ng/ml] | 9.2 (6.3 to 12.7) | 10.5 (7.6 to 13.0) | 24.4 | 9.4 (6.4 to 12.9) | 10.6 (7.6 to 13.0) | 19.6 |
| HOMA2-B | 99.5(59.7 to 148.8) | 120.2 (88.2 to 165.4) | 27.0 | 101.8 (61.2 to 151.1) | 108.4 (77.2 to 153.7) | 9.0 |
| HOMA2-IR | 2.9 (1.8 to 4.9) | 2.1 (1.4 to 3.0) | 49.5 | 2.9 (1.8 to 4.8) | 2.4 (1.6 to 4.0) | 25.7 |
| Hypertension | 163 (94.2) | 565 (76.7) | 51.3 | 146 (93.6) | 147 (94.0) | 1.8 |
| Patients on anti-hypertensive drugs† | 142 (82.1) | 244 (33.1) | 114.1 | 125 (80.1) | 127 (81.6) | 4.0 |
| Total cholesterol [mmol/L] | 4.6 ± 1.1 | 5.0 ± 0.9 | 35.5 | 4.6 ± 1.1 | 5.1 ± 1.1 | 43.0 |
| LDL cholesterol [mmol/L] | 2.6 ± 0.9 | 3.1 ± 0.8 | 56.9 | 2.6 ± 1.0 | 3.1 ± 0.9 | 50.8 |
| HDL cholesterol [mmol/L] | 1.1 ± 0.3 | 1.2 ± 0.3 | 17.9 | 1.1 ± 0.3 | 1.2 ± 0.3 | 26.0 |
| Triglycerides [mmol/L] | 2.0 ± 1.1 | 1.6 ± 1.5 | 30.4 | 2.0 ± 1.2 | 1.9 ± 2.5 | 11.9 |

**Table S1:** ***Patient characteristics in the statin and non-statin treatment groups before and after propensity score-matching.*** Values expressed as numbers (%), mean ± SD or median (IQR) as appropriate. * calculated after handling missing data using multiple imputation procedure. † factors included in the propensity score.

Abbreviations: ASD=absolute standardized difference, BP=blood pressure, HOMA2-B=homeostasis model assessment assessing β-cell function, HOMA2-IR=homeostasis model assessment assessing insulin resistance, HDL=high density lipoprotein, LDL=low density lipoprotein, IQR=interquartile range, SD=standard deviation.

| **Affymetrix Transcript ID** | **log_2_ FC** | **Adjusted P value** | **Gene**  **Symbol** | **Pearson**  **correlation**  **HbA1c** | **P**  **value** | **Pearson**  **correlation**  **HOMA2-IR** | **P**  **value** |
| --- | --- | --- | --- | --- | --- | --- | --- |
| TC01001299.hg.1 | 1.73 | 4.26E-23 | *FDPS** | 2.50E-01 | 7.64E-06 | 8.00E-02 | 2.15E-01 |
| TC07003361.hg.1 | 1.77 | 3.49E-22 | *CYP51A1* | 1.92E-01 | 6.00E-04 | 9.84E-02 | 1.27E-01 |
| TC11003460.hg.1 | 1.39 | 5.92E-22 | *TM7SF2** | 2.64E-01 | 2.09E-06 | 1.38E-01 | 3.20E-02 |
| TC21000554.hg.1 | 1.41 | 1.25E-19 | *LSS** | 2.30E-01 | 3.97E-05 | 1.58E-02 | 8.07E-01 |
| TC05001316.hg.1 | **1.99** | 9.70E-19 | ***HMGCS1*** | 2.80E-01 | 4.58E-07 | 1.54E-01 | 1.64E-02 |
| TC17000319.hg.1 | 1.32 | 5.43E-18 | *TMEM97* | 2.40E-01 | 1.83E-05 | 3.88E-02 | 5.48E-01 |
| TC08000729.hg.1 | 1.87 | 1.34E-17 | *SQLE** | 2.96E-01 | 9.93E-08 | 1.42E-01 | 2.69E-02 |
| TC10000967.hg.1 | 1.45 | 9.22E-17 | *IDI1* | 2.50E-01 | 7.90E-06 | 8.25E-02 | 2.01E-01 |
| TC0X000253.hg.1 | 1.26 | 2.61E-16 | *EBP* | 1.93E-01 | 6.00E-04 | 8.32E-02 | 1.97E-01 |
| TC20000245.hg.1 | 1.36 | 5.06E-16 | *ACSS2* | 2.53E-01 | 5.77E-06 | 5.18E-02 | 4.22E-01 |
| TC06003166.hg.1 | 1.54 | 6.95E-15 | *ACAT2* | 1.44E-01 | 1.06E-02 | 1.97E-02 | 7.61E-01 |
| TC05000363.hg.1 | 1.69 | 1.11E-14 | *HMGCR** | 2.51E-01 | 6.98E-06 | 7.56E-02 | 2.42E-01 |
| TC08000093.hg.1 | 1.36 | 2.78E-14 | *FDFT1* | 2.65E-01 | 1.93E-06 | 7.45E-02 | 2.48E-01 |
| TC0X000743.hg.1 | 1.28 | 1.39E-13 | *NSDHL* | 2.72E-01 | 1.00E-06 | 1.53E-01 | 1.73E-02 |
| TC14001324.hg.1 | 1.34 | 2.16E-13 | *ERG28* | 2.08E-01 | 2.00E-04 | 3.14E-02 | 6.27E-01 |
| TC17002830.hg.1 | 1.16 | 2.39E-13 | *PCYT2* | 2.20E-01 | 8.97E-05 | -7.15E-02 | 2.68E-01 |
| TC11001106.hg.1 | 1.30 | 3.13E-13 | *SC5D* | 1.74E-01 | 2.00E-03 | 2.70E-02 | 6.76E-01 |
| TC07000627.hg.1 | 1.14 | 4.25E-13 | *PCOLCE* | 1.77E-01 | 1.70E-03 | -4.40E-02 | 4.96E-01 |
| TC17001301.hg.1 | 1.32 | 6.86E-13 | *ALDOC* | 2.26E-01 | 5.57E-05 | -1.85E-02 | 7.74E-01 |
| TC16001340.hg.1 | 1.23 | 9.62E-13 | *MVD* | 2.80E-01 | 4.95E-07 | 9.78E-02 | 1.29E-01 |
| TC10000256.hg.1 | 1.52 | 6.40E-12 | *HSD17B7P2* | 3.04E-01 | 4.18E-08 | 9.72E-02 | 1.32E-01 |
| TC22000347.hg.1 | 1.17 | 1.31E-11 | *SREBF2* | 2.44E-01 | 1.31E-05 | 4.22E-02 | 5.13E-01 |
| TC11002038.hg.1 | 1.39 | 1.73E-11 | *DHCR7* | 2.09E-01 | 2.00E-04 | 1.24E-01 | 5.33E-02 |
| TC01001426.hg.1 | 1.49 | 1.73E-11 | *HSD17B7* | 2.95E-01 | 1.10E-07 | 8.10E-02 | 2.09E-01 |
| TC12003078.hg.1 | 1.20 | 3.31E-11 | *MMAB* | 2.41E-01 | 1.69E-05 | -2.90E-02 | 6.53E-01 |
| TC04000828.hg.1 | 1.40 | 1.53E-09 | *MSMO1* | 1.83E-01 | 1.10E-03 | 4.88E-02 | 4.50E-01 |
| TC14002328.hg.1 | 1.25 | 2.70E-09 | *RDH11* | 1.36E-01 | 1.62E-02 | 1.42E-01 | 2.77E-02 |
| TC11003438.hg.1 | 1.18 | 8.01E-09 | *FADS1* | 2.03E-01 | 3.00E-04 | 4.61E-02 | 4.76E-01 |
| TC10000581.hg.1 | 1.15 | 1.07E-08 | *FAM213A* | 1.17E-01 | 3.90E-02 | 1.09E-01 | 9.18E-02 |
| TC0X001506.hg.1 | 1.14 | 1.12E-08 | *CETN2* | 1.95E-01 | 5.00E-04 | 1.55E-01 | 1.58E-02 |
| TC14000604.hg.1 | 1.10 | 1.16E-08 | *AK7* | 2.11E-01 | 2.00E-04 | 6.08E-02 | 3.46E-01 |
| TC06004142.hg.1 | 1.16 | 1.76E-08 | *SOD2* | 9.06E-02 | 1.10E-01 | 7.40E-03 | 9.09E-01 |
| TC16000523.hg.1 | 1.15 | 3.33E-08 | *PDP2* | 1.42E-01 | 1.20E-02 | 8.50E-03 | 8.96E-01 |
| TC06000083.hg.1 | 1.07 | 1.72E-07 | *TMEM170B* | 5.35E-02 | 3.45E-01 | -4.75E-02 | 4.62E-01 |
| TC06001182.hg.1 | **-1.35** | 1.80E-07 | ***UNC93A*** | -1.01E-01 | 7.44E-02 | 6.46E-02 | 3.17E-01 |
| TC07001334.hg.1 | 1.06 | 2.55E-07 | *POLD2* | 1.78E-01 | 1.60E-03 | -3.00E-02 | 6.43E-01 |
| TC05001673.hg.1 | 1.23 | 2.77E-07 | *STARD4* | 1.75E-01 | 1.90E-03 | 6.55E-02 | 3.10E-01 |
| TC21000200.hg.1 | -1.16 | 3.26E-07 | *ABCG1* | -1.81E-01 | 1.30E-03 | -1.48E-01 | 2.09E-02 |
| TC16001701.hg.1 | 1.11 | 7.13E-07 | *SNAI3-AS1* | 1.73E-01 | 2.10E-03 | -4.60E-02 | 4.77E-01 |
| TC12000849.hg.1 | 1.10 | 9.23E-07 | *MVK* | 3.55E-01 | 9.45E-11 | 1.29E-01 | 4.48E-02 |
| TC11000367.hg.1 | 1.16 | 1.58E-06 | *HSD17B12* | 4.69E-02 | 4.08E-01 | 2.85E-02 | 6.59E-01 |
| TC19001502.hg.1 | 1.17 | 2.82E-06 | *CAPN12* | 2.88E-01 | 2.07E-07 | 1.37E-01 | 3.29E-02 |
| TC01000874.hg.1 | 1.17 | 1.36E-05 | *LOC729970* | 1.41E-01 | 1.23E-02 | 1.30E-01 | 4.29E-02 |
| TC17001406.hg.1 | 1.13 | 2.85E-05 | *ACACA** | 1.99E-01 | 4.00E-04 | 1.33E-01 | 3.87E-02 |
| TC07001184.hg.1 | -1.07 | 3.66E-05 | *CDCA7L* | -2.13E-01 | 1.00E-04 | -1.79E-01 | 5.20E-03 |
| TC04001467.hg.1 | 1.20 | 4.25E-05 | *ELOVL6** | 1.15E-01 | 4.26E-02 | -1.45E-02 | 8.22E-01 |
| TC04002196.hg.1 | 1.10 | 4.29E-05 | *LOC100507487* | 1.40E-01 | 1.30E-02 | 1.10E-01 | 8.82E-02 |
| TC0X002327.hg.1 | 1.06 | 6.53E-05 | *PIR* | 2.25E-01 | 5.87E-05 | 8.71E-02 | 1.77E-01 |
| TC11000542.hg.1 | 1.19 | 7.89E-05 | *FADS2** | 1.85E-01 | 1.00E-03 | 6.64E-02 | 3.03E-01 |
| TC06003966.hg.1 | 1.10 | 9.90E-05 | *SOD2-OT1* | 2.02E-02 | 7.22E-01 | -8.80E-03 | 8.92E-01 |
| TC17001192.hg.1 | -1.11 | 2.00E-04 | *SREBF1* | -9.50E-03 | 8.67E-01 | -1.04E-01 | 1.05E-01 |
| TC11003444.hg.1 | 1.06 | 2.00E-04 | *DNHD1* | 1.17E-01 | 3.87E-02 | -6.87E-02 | 2.87E-01 |
| TC01003229.hg.1 | 1.07 | 2.00E-04 | *TDRKH* | 1.89E-01 | 8.00E-04 | 1.27E-01 | 4.86E-02 |
| TC11003443.hg.1 | 1.09 | 2.00E-04 | *TIMM10B* | 1.20E-01 | 3.43E-02 | -7.60E-03 | 9.06E-01 |
| TC07001342.hg.1 | 1.19 | 2.00E-04 | *NPC1L1* | 2.36E-01 | 2.55E-05 | 3.74E-02 | 5.63E-01 |
| TC19000191.hg.1 | 1.18 | 2.00E-04 | *LDLR* | 2.50E-01 | 7.65E-06 | 2.80E-03 | 9.66E-01 |
| TC01003314.hg.1 | 1.07 | 3.00E-04 | *RUSC1-AS1* | 1.84E-01 | 1.00E-03 | 6.04E-02 | 3.50E-01 |
| TC06002088.hg.1 | 1.11 | 3.00E-04 | *ECHDC1* | 1.07E-01 | 5.98E-02 | -7.40E-03 | 9.09E-01 |
| TC16000569.hg.1 | 1.11 | 3.00E-04 | *CYB5B* | 1.02E-01 | 7.21E-02 | 4.68E-02 | 4.69E-01 |
| TC01000129.hg.1 | 1.10 | 4.00E-04 | *PGD* | 1.62E-01 | 4.00E-03 | 1.26E-01 | 5.05E-02 |
| TC21000940.hg.1 | 1.13 | 8.00E-04 | *KCNE1* | 1.10E-01 | 5.29E-02 | -4.62E-02 | 4.75E-01 |
| TC14000454.hg.1 | 1.05 | 1.00E-03 | *BBOF1* | 2.06E-01 | 2.00E-04 | -4.49E-02 | 4.87E-01 |
| TC16002069.hg.1 | 1.09 | 1.00E-03 | *QPRT* | 7.06E-02 | 2.13E-01 | 8.48E-02 | 1.88E-01 |
| TC05001674.hg.1 | 1.10 | 1.40E-03 | *NREP* | -5.48E-02 | 3.34E-01 | -9.80E-02 | 1.29E-01 |
| TC06001692.hg.1 | 1.10 | 1.60E-03 | *GLO1* | -3.40E-03 | 9.53E-01 | 7.20E-03 | 9.12E-01 |
| TC01003565.hg.1 | -1.16 | 2.20E-03 | *ANGPTL1* | -4.93E-02 | 3.85E-01 | -2.73E-01 | 1.64E-05 |
| TC17001973.hg.1 | 1.23 | 2.40E-03 | *FASN** | 2.62E-01 | 2.55E-06 | 1.32E-01 | 4.00E-02 |
| TC15000305.hg.1 | 1.06 | 2.80E-03 | *OIP5-AS1* | -3.19E-02 | 5.74E-01 | -7.20E-02 | 2.65E-01 |
| TC02000751.hg.1 | 1.05 | 3.00E-03 | *DBI* | -4.00E-02 | 4.81E-01 | -4.73E-02 | 4.64E-01 |
| TC19001505.hg.1 | 1.24 | 3.70E-03 | *LGALS4* | 3.06E-01 | 3.19E-08 | 2.93E-01 | 3.63E-06 |
| TC08001556.hg.1 | -1.25 | 4.70E-03 | *ENPP2* | -7.49E-02 | 1.86E-01 | -4.76E-02 | 4.61E-01 |
| TC10000721.hg.1 | 1.27 | 4.80E-03 | *SCD** | 2.26E-01 | 5.42E-05 | 4.90E-02 | 4.48E-01 |
| TC01004184.hg.1 | 1.07 | 7.90E-03 | *NPPA-AS1* | 2.06E-01 | 2.00E-04 | -1.54E-01 | 1.69E-02 |
| TC08000169.hg.1 | 1.04 | 8.70E-03 | *PIWIL2* | 1.28E-01 | 2.33E-02 | -5.84E-02 | 3.65E-01 |
| TC0X001092.hg.1 | 1.05 | 1.19E-02 | *ARHGEF9* | -5.53E-02 | 3.30E-01 | -7.74E-02 | 2.31E-01 |
| TC11003435.hg.1 | 1.03 | 1.21E-02 | *SERGEF* | 1.40E-01 | 1.29E-02 | -6.73E-02 | 2.97E-01 |
| TC05001996.hg.1 | -1.05 | 1.38E-02 | *RNF145* | -9.57E-02 | 9.08E-02 | 1.31E-01 | 4.23E-02 |
| TC03002549.hg.1 | 1.07 | 1.50E-02 | *NUDT16* | -1.05E-01 | 6.30E-02 | -7.06E-02 | 2.74E-01 |
| TC03003123.hg.1 | 1.15 | 1.72E-02 | *PCOLCE2* | -1.38E-01 | 1.49E-02 | -1.06E-01 | 1.01E-01 |
| TC01003847.hg.1 | -1.14 | 1.89E-02 | *DUSP10* | -6.60E-03 | 9.07E-01 | -3.04E-01 | 1.48E-06 |
| TC02000280.hg.1 | -1.08 | 2.03E-02 | *PRKCE* | 6.80E-03 | 9.04E-01 | 2.45E-01 | 1.00E-04 |
| TC01002307.hg.1 | -1.20 | 2.12E-02 | *PLA2G2A* | -1.69E-02 | 7.66E-01 | -8.70E-03 | 8.93E-01 |
| TC03000773.hg.1 | 1.05 | 2.16E-02 | *PAQR9-AS1* | -6.60E-03 | 9.07E-01 | 1.22E-02 | 8.50E-01 |
| TC12001003.hg.1 | 1.04 | 2.30E-02 | *AACS* | 2.42E-01 | 1.49E-05 | 1.89E-01 | 3.20E-03 |
| TC05002041.hg.1 | 1.10 | 2.47E-02 | *PANK3* | 1.05E-01 | 6.29E-02 | -1.95E-02 | 7.63E-01 |
| TC17002528.hg.1 | 1.06 | 2.49E-02 | *SHMT1* | 4.25E-02 | 4.54E-01 | 6.05E-02 | 3.48E-01 |
| TC17002142.hg.1 | -1.05 | 2.55E-02 | *SLC13A2* | -5.20E-03 | 9.27E-01 | -5.67E-02 | 3.80E-01 |
| TC01001781.hg.1 | 1.07 | 2.59E-02 | *FLVCR1* | 2.58E-01 | 3.62E-06 | 9.61E-02 | 1.36E-01 |
| TC21000257.hg.1 | 1.06 | 2.61E-02 | *MCM3AP-AS1* | 1.95E-01 | 5.00E-04 | 8.28E-02 | 1.99E-01 |
| TC03001466.hg.1 | 1.05 | 2.68E-02 | *TKT* | 2.82E-01 | 3.93E-07 | 8.78E-02 | 1.73E-01 |
| TC14000401.hg.1 | 1.09 | 2.88E-02 | *GPHN* | -4.25E-02 | 4.53E-01 | -1.16E-01 | 7.18E-02 |
| TC11003343.hg.1 | 1.05 | 3.28E-02 | *TIMM8B* | 9.14E-02 | 1.07E-01 | 1.62E-01 | 1.16E-02 |
| TC06001072.hg.1 | 1.11 | 3.43E-02 | *SAMD5* | 1.87E-02 | 7.42E-01 | -1.16E-01 | 7.26E-02 |
| TC09000668.hg.1 | 1.05 | 3.95E-02 | *STXBP1* | 3.02E-01 | 4.84E-08 | 1.03E-01 | 1.10E-01 |
| TC01002694.hg.1 | 1.10 | 4.00E-02 | *DHCR24* | 5.00E-04 | 9.93E-01 | -4.70E-03 | 9.42E-01 |
| TC04000829.hg.1 | -1.10 | 4.33E-02 | *CPE* | 7.90E-02 | 1.63E-01 | 9.38E-02 | 1.46E-01 |
| TC10000010.hg.1 | 1.03 | 4.41E-02 | *WDR37* | -1.10E-03 | 9.84E-01 | -1.44E-01 | 2.52E-02 |
| TC12001155.hg.1 | -1.12 | 4.51E-02 | *LPCAT3* | -3.54E-02 | 5.33E-01 | -5.93E-02 | 3.59E-01 |

**Table S2A: *Differentially expressed genes between statin and non-statin treated patient groups from the matched patient cohort with Pearson correlation parameters to HbA1c and HOMA2-IR.*** Genes were ranked according to the corrected p value. *selected genes for validation in quantitative polymerase chain reaction (qPCR) assays (Supplemental Table 3). Abbreviations: FC=fold change. Red, bold characters: highest upregulated gene; green, bold characters: highest downregulated gene.

|  | **Without adjustment for diabetes status** | |  | **With**  **adjustment for diabetes status** | |
| --- | --- | --- | --- | --- | --- |
| **Affymetrix Transcript ID** | **log_2_ FC** | **Adjusted P value** | **Gene**  **Symbol** | **log_2_**  **FC** | **Adjusted**  **P value** |
| TC01001299.hg.1 | 1.73 | 4.26E-23 | *FDPS* | 1.70 | 7.77E-21 |
| TC07003361.hg.1 | 1.77 | 3.49E-22 | *CYP51A1* | 1.38 | 8.93E-20 |
| TC11003460.hg.1 | 1.39 | 5.92E-22 | *TM7SF2* | 1.41 | 1.42E-19 |
| TC21000554.hg.1 | 1.41 | 1.25E-19 | *LSS* | 1.40 | 9.82E-18 |
| TC05001316.hg.1 | **1.99** | 9.70E-19 | ***HMGCS1*** | **1.91** | **4.25E-16** |
| TC17000319.hg.1 | 1.32 | 5.43E-18 | *TMEM97* | 1.31 | 9.45E-16 |
| TC08000729.hg.1 | 1.87 | 1.34E-17 | *SQLE* | 1.79 | 6.77E-15 |
| TC10000967.hg.1 | 1.45 | 9.22E-17 | *IDI1* | 1.41 | 1.63E-14 |
| TC0X000253.hg.1 | 1.26 | 2.61E-16 | *EBP* | 1.26 | 1.15E-14 |
| TC20000245.hg.1 | 1.36 | 5.06E-16 | *ACSS2* | 1.35 | 3.28E-14 |

**Table S2B: Top 10 *differentially expressed genes between statin and non-statin treated patient groups from the matched patient cohort with and without adjustment for diabetes status.*** Genes were ranked according to the corrected p value. Abbreviations: FC=fold change. Red, bold characters: highest upregulated gene.

| **Affymetrix Transcript ID** | **Log_2_FC (Affymetrix)** | **Adjusted P value (Affymetrix)** | **FC (qPCR)** | **P value (qPCR)** | **Gene Symbol** |
| --- | --- | --- | --- | --- | --- |
| TC11003460.hg.1 | 1.4 | 5.94E-22 | 1.9 | <0.0001 | *TM7SF2 ** |
| TC01001299.hg.1 | 1.7 | 4.26E-23 | 1.9 | <0.0001 | *FDPS ** |
| TC21000554.hg.1 | 1.4 | 1.25E-19 | n.d. |  | *LSS ** |
| TC08000729.hg.1 | 1.9 | 1.34E-17 | 2.9 | <0.0001 | *SQLE ** |
| TC05000363.hg.1 | 1.7 | 1.11E-13 | 2.6 | <0.0001 | *HMGCR ** |
| TC04001467.hg.1 | 1.2 | 6.91E-09 | 1.6 | <0.0001 | *ELOVL6 *** |
| TC11000542.hg.1 | 1.2 | 6.89E-05 | 1.0 | n.s. | *FADS2 *** |
| TC10000721.hg.1 | 1.3 | 4.80E-03 | 1.6 | <0.0001 | *SCD *** |
| TC17001406.hg.1 | 1.1 | 2.85E-05 | 1.1 | n.s. | *ACACA *** |
| TC17001973.hg.1 | 1.2 | 2.40E-03 | 1.5 | <0.05 | *FASN *** |

**Table S3: *Q-PCR validation* *of 10 statin-regulated genes identified from the Affymetrix microarray analysis.*** Selected genes are part of cholesterogenic (SREBP2- regulated *) and lipogenic (SREBP1-regulated **) pathways and were measured in liver samples from statin-treated (n=40) and non-statin treated (n=40) patients. Abbreviations: FC=fold change, n.d.=not detectable, n.s.=not significant.

| **Pathway or Disease** | **# Genes** | **Statin-regulated genes (Gene Symbols)** |
| --- | --- | --- |
| Superpathway of cholesterol biosynthesis | 19 | *ACAT2,CYP51A1,DHCR7,DHCR24,EBP,FDFT1,FDPS,HMGCR,HMGCS1,HSD17B7,IDI1,LSS,MSMO1,MVD,MVK,NSDHL,SC5D,SQLE,TM7SF2* |
| Fatty acid metabolism | 18 | *AACS,ABCG1,ACACA,ACSS2,DBI,ELOVL6,FADS1,FADS2,FASN,LSS,MSMO1,NPC1L1,PCOLCE2,PLA2G2A,SC5D,SCD,SREBF1,STARD4* |
| LXR/RXR signaling | 8 | *ABCG1,ACACA,CYP51A1,FASN,FDFT1,HMGCR,SCD,SREBF1* |
| AMPK signaling | 4 | *ACACA,AK7,FASN,HMGCR* |
| Metabolic disease | 31 | *AACS,ABCG1,ACACA,ACAT2,CPE,CYP51A1,DHCR7,DHCR24,EBP,FADS2,FASN,FDFT1,FDPS,GPHN,HMGCR,KCNE1,LSS,MMAB,MSMO1,MVK,NPC1L1,PLA2G2A,PRKCE,QPRT,SC5D,SCD,SLC13A2,SOD2,SREBF1,SREBF2,STXBP1* |
| Type 2 diabetes mellitus | 15 | *AACS,ABCG1,ACACA,FADS2,FASN,FDFT1,HMGCR,KCNE1,NPC1L1,PRKCE,QPRT,SCD,SOD2,SREBF1,SREBF2* |
| Insulin resistance | 10 | *ACACA,ACAT2,CPE,FASN,HMGCR,NPC1L1,PRKCE,QPRT,SCD,SREBF1* |

**Table S4:** ***Pathway assignment to statin-dysregulated genes.*** The list of genes from Supplemental Table 2 were assigned to main metabolic and signaling pathways or disease processes using the Ingenuity Pathway Analysis (IPA). Abbreviations: LXR/RXR=liver X receptor/retinoid X receptor, AMPK=AMP-activated protein kinase.
